# Do Access to Finance, Technical Know-How, and Financial Literacy Offer Women Empowerment Through Women’s Entrepreneurial Development?

**DOI:** 10.3389/fpsyg.2021.776844

**Published:** 2022-01-04

**Authors:** Anselme Andriamahery, Md. Qamruzzaman

**Affiliations:** ^1^School of Economics and Management, Hubei Polytechnic University, Huangshi, China; ^2^School of Business and Economics, United International University, Dhaka, Bangladesh

**Keywords:** access to finance, technical know-how, financial literacy, women empowerment, entrepreneurial sustainability

## Abstract

The motivation of the study is to gauge the effects of access to finance, technical know-how, and financial literacy on women’s empowerment through establishing women’s entrepreneurial development. A sample of 950 women-owned SMEs was considered, and structured questionnaires were sent from getting target responses. After careful assessment through the data cleansing procedure, it was found that only 795 responses are suitable for further investigation, implying the sample response rate for the study is 74.71%. The study implemented structural equation modeling and multivariate regression analysis for gauging the causal association that is direct and indirect effects of target variables. According to findings, a positive statistically significant linkage was revealed with women’s entrepreneurship sustainability and women empowerment. Furthermore, the mediating effects were also established for women’s empowerment. According to the study findings, it is suggested that for women entrepreneurship sustainability, effective policies surrounding financing accessibility, technical knowledge expansion, and financial understating have to be promulgated in the economy, which allows bringing women empowerment at large.

## Introduction

Women’s empowerment is a major issue in global development; despite their great contribution to development, women face discrimination, especially in developing countries. Women are also treated worse than men owing to society’s rules, norms, customs, and character. This unreasonable attitude toward women puts them at a disadvantage socially, culturally, religiously, economically, and legally. Furthermore, due to a paucity of resources and an overcrowded country, Bangladeshi women are the most disadvantaged, and services and opportunities are strictly separated by gender, class, and area. The study of Khanum et al. (2020) postulated that women’s economic empowerment is critical for sustainable development because, without their involvement in mainstream development programs, it would be impossible to institutionalize the sustainable development process in Bangladesh. Since the 1990s, Bangladesh has experienced a boom in women entrepreneurs and their contribution to the economy. Women entrepreneurship growth is required to empower females and help them better their financial situation. By encouraging women to start and operate their businesses, women SMEs improve their financial potential. It is also the key driver of progress in underdeveloped nations like Bangladesh (Zafar and Mustafa, 2017).

Bangladesh is likewise making strides toward achieving middle-income status by 2021 when the country’s per capita income will reach US$ 3500. Bangladesh made great achievements in women and child development efforts from 2008 to 2018, particularly in women empowerment, women’s decision-making, health and nutrition, and small business growth, including job creation. These achievements in socio-economic areas enlarged and expanded the window of opportunity for attaining gender equality. Bangladesh was ranked #1 for the second straight year in the World Economic Forum’s Gender Gap Index 2017. Womenfolk in Bangladesh are increasingly acting as catalysts for development activities. Thus, Bangladesh has paid pragmatic attention to economic development, educational development, and social changes that improved living standards and provided health care to the common people. Most precisely to empower half of the country’s neglected population, that is, women. Bangladesh cannot achieve its long-term development goal without women’s empowerment. As a result of women’s empowerment, the government has implemented national policies to assist the growth of women in all fields of economic activity, with a particular emphasis on their entrepreneurial development.

Women entrepreneurs have evolved with SMEs over the past decade, and Bangladesh’s government has emphasized women’s development by offering targeted policy formulation and implementation. 55.8% of females in Bangladesh exist beneath the poverty line (UNDP, 2015) and trying hard to drag out from the vicious poverty cycle through capitalizing on entrepreneurial skills. Moreover, women’s entrepreneurial development ensures the excess capacity to increase household earnings and pay for primary education, clothing, and food (Nasir et al., 2019). Women establish and run businesses in different sectors than males, which means they have lower involvement rates in entrepreneurship.

The contribution of the study in the existing literature is as follows. First, women’s entrepreneurship development and women empowerment has been investigated in literature and revealed a strong correlation (Nawaz, 2010; Dwivedi and Dwivedi, 2011; Morshed and Haque, 2015). It implies that women’s empowerment, which is the capacity to increase purchasing power with economic stability, is a noticeable outcome of women’s entrepreneurship development. The evolvement of women’s entrepreneurship in Bangladesh has gotten attention from researchers, policymakers, and donor agencies, and several policies have been initiated for entrepreneurship development among women participants. However, the empirical literature has not extensively investigated the association between women’s entrepreneurship development and empowerment. To our best knowledge, this is the first-ever empirical assessment to explore the fresh insight regarding the nexus between women’s entrepreneurship development and women empowerment.

Second, the study extends the existing literature regarding the determinants of women empowerment and entrepreneurial development by taking technological know-how and financial literacy into the equation. Even though empirical studies have tried to detect the key determinants for women’s entrepreneurship development and empowerment, the mediating effects of access to credit, technical know-how, and financial literacy have yet to unleash.

Third, the study will be helpful to understand the issues which women are facing in empowering themselves in entrepreneurship. There are many factors discussed in this study that are helpful for policymakers to understand the necessity to improve this area. Women will be involved in the business, and they could be more empowered and self-influencing behavior. They can take part in leading any organization or solve any issue of their lives. The study will be beneficial to pursue the other adapts the empowering behavior in any stage of their lives. Moreover, the study can add value to women’s entrepreneurship, leading to empowering behavior toward economic growth in Bangladesh. Further, it can be adding value in the education sector to training in education for enhancing the empowering behavior to survive with social norms in a conservative culture (Datta and Gailey, 2012).

The motivation of the study is to explore the role of access to finance, technical know-how, financial literacy in the process of women empowerment in Bangladesh through the development of women entrepreneurial development. To test the prospective association, the study investigated both direct and indirect effects on women’s empowerment. Refers to study findings, the tested hypothesis has proved valid regarding the direct contributions from access to finance, financial literacy, and technical know-how in women empowerment in Bangladesh.

The remaining structure of the manuscript is as follows. Section 2 deals with the literature survey and theoretical development of the study. Data, variables definition, and estimation strategy are explained in Section 3. Section 4 contains data analysis and interpretation. Discussion of the results displayed in Section 5 and conclusion and policy recommendations are available in Section 6.

## Theoretical Development and Literature Survey

### Theoretical Development

Network Affiliation theory proposes that entrepreneurship is “entrenched in a complex network of social relations.” According to this point of view, entrepreneurship is facilitated or limited inside this community through connections between aspiring entrepreneurs, assets, and opportunities. The existence or absence of these professional relations affects the entrepreneur’s performance (Muteru, 2013). This theory perceives that network availability, like contribution in relations, assumes an essential function in impacting business people’s exhibitions. Just as business people who are women have phenomenal individual and interpersonal organizations than men, their entire exhibition in enterprising exercises subsequently stays extraordinary. Consequently, more often than not, the need of a woman is family government assistance rather than standard taking the enterprising capacity. Women may find it trying to utilize and control work, to work out the imperative purpose of portability (Kabeer, 2012).

By engaging women using business, improvement has become addressed because the real women’s strengthening alludes to women’s ability to practice inclinations concerning the three interconnected elements of assets, business undertaking (cycle), and accomplishments. Thus, it is required that to make particular the strengthening of women, and they should have the availability to consummate informal organization, for example, land, training, an opportunity of inclination in making any determination excepting having any duplicity (measure) lastly gets the achievement other than any pressure. Consequently, because of the previously mentioned thoughts, the hypothetical structure of this investigation inspects the issues and conceivable outcomes of money-related strengthening of women through business enterprise advancement in Bangladesh with a point of convergence on the methodologies over which a lady transforms into an SME business person in the nation. It will help find the difficulties lady faces while in transit to become a business visionary in SMEs in Bangladesh to procure this examination’s goal. Subsequently, this paper investigates why and how women cooperate with SMEs and their difficulties in unique degrees of SME organizations dependent on the accompanying system.

Furthermore, the study also considered the resources-based theory and pecking order theory to conceptualize model assessment in gauging the association between access to finance, technical know-how, and financial literacy on women entrepreneur sustainability and empowerment. The Resource-Based Theory (RBT) is adapted in this study to explain the relationship between the dependent variable and the mediating variable performance of women empowerment as the dependent variable, and the mediating role of women entrepreneurship sustainability in exploiting the strengths and weaknesses of businesses, which may result in outperformance. Access to financing, technical know, and financial literacy are used as independent variables in this research.

While the Pecking Order Theory (POT) was developed in response to a lack of asymmetric knowledge about financial markets and high transaction costs associated with external borrowing, in contrast to external investors, the idea asserts that managers are often the only custodians of a substantial portion of knowledge about their companies’ circumstances and prospects. The research is mainly concerned with how companies create and accomplish their results. According to POT, funding sources should be chosen in a hierarchical order. Thus, women entrepreneurs who face a funding crisis seek funding from friends, family, and families and retain earnings to protect assets, loans, and agencies to enhance their performance.

### Literature Survey and Hypothesis Development

#### Women Entrepreneurship and Women Empowerment

Women entrepreneurs are critical to sustainable economic growth and social progress because of the emergence of globalization. Moreover, women have been seen as new engines of development. Women entrepreneurs have been labeled as the next rising stars of the economy in emerging nations, potentially increasing wealth and welfare (Darzi et al., 2016). Women entrepreneurship initially seemed in the literature of entrepreneurship for greater than 30 years back. The research in women’s entrepreneur is now directed around the world by various scholars (Jennings and Brush, 2013). Various research exhibit that entrepreneurship is mostly men-dominated in the environment; however, no matter that, entrepreneurship is regularly seen as a form of a woman. Business enterprise encourages women’s self-empowerment *via* giving independence, opportunity, self-assurance, and creativity (Gill and Ganesh, 2007). Women empowerment is defined as women’s involvement in the workforce, leadership roles in social and political problems, and access to credit (Sarker, 2006). Women’s empowerment has lately received significant attention as a policy problem in most world organizations. With their cheap capital requirements and huge job creation potential, small businesses may act as catalysts for economic growth by breaking the vicious cycle of poverty (Shrivastava, 1994).

Women empowerment has arisen as a critical topic in the last several years. Nowadays, women’s economic empowerment is seen as a prerequisite for a country’s sustainability. The capacity of women to participate in, contribute to, and profit from growth processes is sometimes referred to as economic empowerment (Eyben et al., 2008). To put it another way, therefore, the challenge of giving women more economic empowerment is of vital significance to political philosophers, social scientists, and reformers. There are employment, financial services, property, skills development, and market knowledge available to economic empowerment women.

A study conducted by Khanum et al. (2020) investigates the women’s entrepreneurship effects on empowerment in Bangladesh by employing descriptive statistics considering 160 respondents. The study documented that access to external finances, among other factors, plays a prominent role in women’s entrepreneurial development. The economic opportunities for females are resulting from the enterprise support different sorts of strengthening of females in the general public that prompt the disposal of gender gap and protection (Banihani, 2020). Different kinds of support comprise females’ social and political support, and all acts of strengthening are associated with one another. It might be battled that the advancement of business among females is a positive method to recover an awful economy. “Among different kinds of a business task, women organizations are a principal zone in adding to budgetary and task to improve helpless females for their outstanding (Nasir et al., 2019). According to literature, dominated society has also restricted women from growing their businesses (Parker et al., 2007).

H1: *Women entrepreneurship in SMEs has a positive effect on women’s empowerment.*

#### Mediation Effect of Women Entrepreneurship Between Access to Finance and Women Empowerment

Women entrepreneurs, especially in underdeveloped nations, struggle to obtain financial resources, resulting in instability for women’s entrepreneurial development in the formal sector, while women outnumber men in the informal economy (Ike, 2013). Women entrepreneurs in underdeveloped nations, like Nigeria, have unique challenges in obtaining financial resources (Vuong, 2016). A lack of formal education and early marriage are cultural barriers women face since their husbands are too busy to assist at home, even when needed. Furthermore, males do not allow their wives to leave the house due to severe religious and cultural restrictions (Idris and Agbim, 2015).

Women entrepreneurship development, sustainability, and empowerment, whatever the cases, the limited access to external credit facility has revealed one of the critical attributes in literature (De Vita et al., 2014; Aliyu et al., 2019; Alene, 2020). The capacity to acquire financial services, such as credit, deposit, payment, insurance, and other risk management services, is crucial in operational expansion and financial stability (Demirgüç-Kunt et al., 2008). Enterprises’ progress will be influenced by a broadening of the Access to financing effective asset allocation, exploiting growth opportunities, and increased company innovation and dynamism are all connected to a growth mindset. Access to capital increases the likelihood of a company entering the market and growing to a bigger size, enabling the firm to take advantage of more efficient legal forms and conduct operations on a larger scale.

According to Adesua-Lincoln (2011), credit availability is the central element influencing entrepreneurs’ start-up and development. The study found that financial resources and collateral most limit women entrepreneurs in Nigeria. When it comes to funding their start-ups, most women entrepreneurs rely on internal sources of money. The availability of bank credit appears the organization to become developed or conceivably. Mostly Banks are not relying on women to provide the credit in business initiatives while they demand some securities to give loans. In a study, Khaleque (2018) documented a positive linkage between access to finance and operational performance in women entrepreneurs in Bangladesh. In the same vein, Chamani et al. (2017) suggested that credit availability can be helpful to increase women’s entrepreneurship. Taiwo et al. (2016) investigated the effect of credit facilities on women entrepreneurs on job creation for women in Nigeria. According to the study funding women, entrepreneurs is favorably linked with job creation through increasing their company operations. Another issue recognized by several surveys is difficulties in access to finance. Many financing institutions have failed to provide loan facilities. Banker’s behaviors should be helpful for women in business development. The result of the booming business of women enhances women empowerment (Vuong et al., 2021).

Women are less likely to get credit than males for many reasons, including a lack of collateral, reluctance to use household assets as security, and the unprofitable nature of the credit they apply for, which means formal financial organizations do not provide it to them (Abd Wahab and Abdesamed, 2012).

H2: *Women entrepreneurship has a positive mediating effect between access to finance and women empowerment*

#### Mediation Effect of Women Entrepreneurship Between Financial Literacy and Women Empowerment

Financial educational programs aiming at increasing financial literacy educate people about various financial ideas and instruments and empower them by improving their financial abilities related to the usage of financial goods and risk management. Gupta and Kaur (2014) advocated that financial skills help people improve their financial literacy levels. In another study, Finke et al. (2017) postulated that financial literacy is the possession of financial knowledge and ability to manage money and enables the capacity to use knowledge and skills to manage financial resources for a lifetime of financial wellbeing properly.” Financial literacy is also described by Engström and McKelvie (2017) in terms of financial knowledge and abilities. Financial literacy encompasses understanding financial ideas and using such information to make good financial choices and create methods for successfully managing financial resources (Lusardi and Tufano, 2015).

Entrepreneurs often act instinctively, take calculated risks, and occasionally, due to their overconfidence and optimism, fail to evaluate all of the information shown to them about the company they have chosen, and therefore suffer several losses. Financial literacy is critical for entrepreneurs because it equips them to make sound judgments based on facts and make the most prudent financial choices. Entrepreneurship and financial literacy are topics high on the worldwide agenda since they facilitate the effective use of financial resources. Adequate financial education promotes prudent product selection based on educated judgments (Atandi et al., 2017). In literature, several studies have been performed in assessing the role of financial literacy on entrepreneurial development across the world see Kojo Oseifuah (2010); Atandi et al. (2017), Cossa et al. (2018); Egbo et al. (2020), and Nguyen et al. (2021) among others.

A study conducted by Egbo et al. (2020) investigates the association between financial literacy and access to credit by women entrepreneurs in Nigeria. The study documented that financial knowledge was a key element in expanding women-owned businesses, particularly during start-up. Additionally, the study established that financial expertise is essential to the development and success of women-owned businesses. Financial literacy and ability are necessary for women to build an entrepreneurial spirit and grow their entrepreneurial activities effectively, manage their personal and family money, and increase their success in their entrepreneurial areas of interest. It is critical to equip women entrepreneurs with the knowledge and skills necessary to make smart and good financial decisions. Sucuahi (2013) disclosed that financial literacy positively augments the growth prospects of SMEs by enabling the owners for efficient funds management and appropriate credit sources selection. The study also postulated that women’s entrepreneurial growth immensely relies on appropriate credit selection.

Usama and Yusoff (2018) have investigated the relationship between entrepreneurial financial literacy on firm performance and sustainability in women entrepreneurship in Nigeria. Study findings unveiled that financial skills and firms’ sustainability move together: business empowerment through appropriate credit selection and funds maximization allows higher profitability with lower financing cost. Thus, the study emphasized enhancing financial literacy through financial understanding, which eventually assists them in running successful operations. Financial literacy refers to the extent to which an individual comprehends critical financial concepts and has the ability and confidence to manage money prudently, focusing on short-term decision-making and long-term financial planning (Remund, 2010).

H3: *Women entrepreneurship has a positive mediating effect on financial literacy and women empowerment*

#### Mediation Effect of Women Entrepreneurship Between Technological Skills and Women Empowerment

The emergence of women entrepreneurs and their contribution to the national economy has been critical for a growing country. Women have begun to contribute to economic development via their unique professions. They have evolved from their traditional roles as housewives into significant business figures with the capacity and passion for managing a company independently and confronting any challenges that may arise to benefit from it. Women entrepreneurs now have additional possibilities to develop their companies and become more successful due to the expanding IT environment. IT is also one factor that is recognized as more important in decision-making and organizations’ activities: how it can be possible to conceptualize and benchmark work. It helps empower women entrepreneurship’s good behavior in a particular culture and society (Vossenberg, 2013).

Information technologies have long been present in many aspects of human life and hugely affect business dealings. Mobile phones, computers, and the Internet are essential for company growth and market competitiveness. IT has several uses in enterprises, from facilitating communication between parties to online sales. IT systems impact the firm’s goods, markets, costs, and product differentiation. Thus, the creative use of IT is essential to the success of innovative companies. Prljić et al. (2015) performed an investigation to explore the role of technological skills on women’s entrepreneurial success and performance. The study revealed that technological know-how produces an edge for women entrepreneurs by acquiring advanced knowledge and ensuring operational efficiency.

According to Costanza et al. (2003), women’s entrepreneurship contributes to economic development and provides job possibilities. Various pieces of research have indicated a link between women entrepreneurship and women empowerment. Empowerment is the process by which an individual moves from a position of powerlessness to one of relative control over his or her life, destiny, and surroundings. This shift may be seen in both an increase in perceived capacity to control and an increase in actual ability to control.

H4: *Women entrepreneurship has positive mediating effect on technological skills and women empowerment*

## Research Sample and Instruments

### Research Design and Sample

According to BBS and SME Foundation, approximately 2.5 million women entrepreneurs were doing business in the economy of Bangladesh, with an approximate growth rate of 11.25% for the period 2010–2020. However, for study purposes, 950 women-owned SMEs were considered the population frame based on their locational presence, Dhaka city. The study only considered those women-owned SMEs doing business over the last 5 years and located in the data city. Almost 60% of women entrepreneurs in Bangladesh adopted their traditional businesses like parlors, Boutiques, etc. The examination utilized purposive sampling to choose respondents, and dependent on the most accessibility of the members was chosen. Data collected using the questionnaire enabled the scientist to achieve a large sample within a constrained time. Gall and Borg (1989) see that the questionnaire utilized descriptive data from a large sample. It also claims the objective response because of its confidentiality. Structured questionnaires were used in this study, while the structured questionnaire was easy to fill from respondents. Dhaka city was picked in light of the maximum accessibility of women SME entrepreneurs compared to other cities. Structured questionnaires were prepared to get responses from target respondents, and research assistance personally visited their establishments and accumulated their responses. The entire data collection activity was imitated and completed from March 14, 2021, to July 26, 2021. After careful consideration and data cleansing procedure, it was revealed that several responses should not be considered for further assessment due to missing value and inconsistent feedback. Altogether, 795 responses were fund suitable for assessment which is about 93.87% of the total target sample.

Table 1 shows the technical details of responses.

**TABLE 1:** Technical details for research sample.

Category	Remarks
Sector	Women-owned SMEs
Geographical location	Bangladesh (Dhaka)
Methodology	Random sampling
Population (sample)	950 (796)
Data collection period	March 14, 2021, to July 26, 2021

*Sources: Authors accumulation (2021).*

### Measurement Model

**FIGURE 1:**
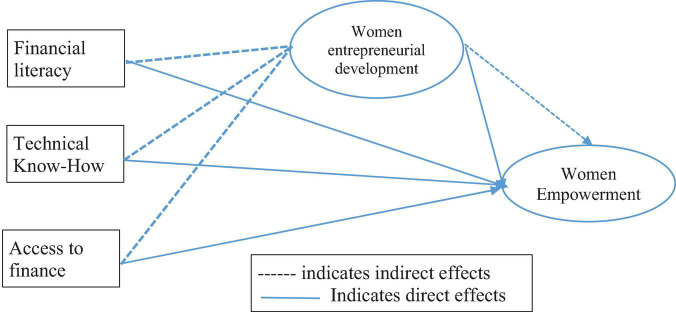
Conceptual framework women empowerment through entrepreneurship in Bangladesh.

The study performed three different causal models that are first model leading to exploring the role of access to finance, technical know-how and financial literacy on women entrepreneurial development, second model dealing with the detection of women empowerment with access to finance, technical know-how, and financial literacy, and finally the full model which is dealing with mediating role of women entrepreneurial development in the process of women empowerment. The proxy indicators for each variable in the measurement model are displayed in Table 2 and the conceptual model display in Figure 1.

**TABLE 2:** Measurements of variables.

Variables	Definition	Latent construct	References	Scale for response
Women empowerment (*WoE*)	It is all about power – the capacity to redefine our possibilities and choices and the power to act on them. It is about individuals using their ability to be courageous and believe in themselves	6	Mehra et al., 2012; Hunt and Samman, 2016	Disagree 1 to strongly agree 5
Women entrepreneurship development (*WeD*)	It is referred to as skills development with innovation, managerial competency, and technical know-how for managing enterprises.	5	Ming Yen Teoh and Choy Chong, 2014; Abd Rani and Hashim, 2015	
Access to finance (*AoF*)	Access to finance is the ability of individuals or enterprises to obtain financial services, including credit, deposit, payment, insurance, and other risk management services	5	Oyewale et al., 2013; Pu et al., 2021	
Technical Know-how (*TkH*)	technical opportunities: scientific knowledge, technological advancements	5	Prljić et al., 2015	
Financial literacy (FiL)	Knowledge, behavior, attitude, and skills	4	Buallay et al., 2017; Hamdan et al., 2021	

#### Women Empowerment

Women empowerment (referred to as WoE in Tables) is a multidimensional term that manifests differently in various cultures and contexts. There is a substantial body of research on the subject, and agreement exists that women empowerment is a process including resources, agency, and accomplishments (Buvinic and Furst-Nichols, 2014). Empowerment, also known as “exercise of agency,” is a process that results in a final women empowerment outcome that has both an objective (economic accomplishment) and a subjective component (economic empowerment). The main aspects of women empowerment may be classified as contextual and household variables that influence women’s economic opportunities and capacities, including individual (and community) endowments that allow women to exercise agency and capitalize on economic possibilities. Resources refer to education strength, financial future security, and wealth for investment (Corbridge, 2002).

Furthermore, access to resources has greater potential to accessibility in allocation and execution of expectations (Sen, 2014). Agency explained that Being able to influence significant aspects of one’s life via one’s actions and the ability to make and act on choices and control resources and earnings is known as economic empowerment (Mehra et al., 2012; Buvinic and O’Donnell, 2017). Achievements stand for wellbeing and sustainable progress.

#### Women Entrepreneurial Development

Conflicts between work and family hinder women from participating in entrepreneurship as much as males (Abd Rani and Hashim, 2015). Because of it, women are often thought to have fewer chances of success when they start their businesses. Various organizations aimed at helping women entrepreneurs have been created to serve as a forum for them to expand and strengthen their network, to provide training courses, seminars, and ceremonies to empower and inspire them (Shobirin et al., 2003). In describing women’s studies, there are many variations on the definition of success other than the measurements, which vary greatly (Lai et al., 2010; Teoh and Chong, 2014). Alam and Yasin (2010) pointed out that, unlike men entrepreneurs, women entrepreneurs do not consider their financial success if their businesses have slower development and smaller size. They believe that the success of women business owners is defined by how well they manage their many areas of life.

#### Access to Finance

Access to finance refers to the applicant’s ability to obtain credit, savings, and insurance services quickly and easily. As a result, eligible women entrepreneurs who perceive and use financial institution credit services are considered to have access to funding (Okello Candiya Bongomin et al., 2017).

#### Technical Know-how

Technological knowledge is a broad term that encompasses knowledge about goods, technology, and processes (Burgers et al., 2008). The accumulation of technical knowledge allows more effective use of related information, but it also helps companies better comprehend and assess the nature and economic potential of technological advancements (Cohen and Levinthal, 1990). Klevorick et al. (1995) identified three primary sources of technical opportunities: scientific knowledge, technological advancements in other sectors, and positive feedback from past periods of technological advancement.

#### Financial Literacy

Financial literacy is critical for financial inclusion and is often seen as the first step toward attaining it (Shusha, 2017). Individuals must grasp the fundamentals of money management to financially empower themselves and improve their overall wellbeing (Arofah et al., 2018). Governments are promoting financial inclusion because numerous studies, such as those conducted by Chibba (2009), and Hussaini and Chibuzo (2018), have demonstrated that financial inclusion provides complementary and incremental solutions for poverty reduction and achieving the Millennium Development Goals. Financial literacy assists individuals in making financial choices. Informed financial decisions on investments, savings, banking, and borrowing are less susceptible to financial fraud. The complexity of financial goods readily accessible to broad audiences, such as internet banking, e-wallets, etc., has raised the significance of financial expertise. Globally, governments strive for financial inclusion to provide access to financial services, including bank accounts and credit products. Moreover, improvements in financial goods have empowered individuals who previously depended on their jobs or governments for financial security (Klapper et al., 2015). Furthermore, financial illiteracy exposes individuals to risks and expenses since they end up in greater debt, pay more interest, accept high rate credit, borrow more, and save less (Lusardi and Tufano, 2015).

Financial literacy, according to Gupta and Kaur (2014), is a collection of financial knowledge, financial awareness, financial skill, attitude, and behavior that a person must possess in order to make sound financial choices and attain financial wellbeing. Remund (2010) discovered that financial literacy is comprised of five domains: financial concept knowledge, the ability to comprehend and communicate about financial concepts, a financial attitude and aptitude for managing personal finances, financial skills necessary to make sound financial decisions, and an individual’s confidence in making sound financial decisions. Thus, Arofah et al. (2018) define financial literacy as people learning a set of abilities in various areas, including statistical literacy, computer literacy, and health literacy (2018).

### Descriptive Statistics

The descriptive assessment of the research unit is displayed in Table 3, and it is manifested that 74.58% of women entrepreneurs represent services-focused business activities, and 25.16% deal with manufacturing activities. 35.22% of SMEs reports their annual revenue between 5–10 million and 55.35% of entrepreneur reports annual average revenue less than 5 million, and 9.13% of SMEs have shown average earning between 10 to 15 million. According to employment concentration, it is revealed that about 22.01% of SMEs were operating with 150 human resources, between 25–150 employes (35.85%) and fewer than 25 employes (42.14), suggesting women entrepreneur operations predominately rely less on human resources due to concentrated on small scale operation.

**TABLE 3:** Demographic profiles of research units.

Classification		Total	%	Samples
Nature of business	Manufacturing Units	200	25.16%	795
	Services units	595	74.84%	
Yeas of establishment	less than 10	45	5.66%	795
	between 10 to 15 years	228	28.68%	
	between 15 to 20 years	225	28.30%	
	more than 20 years	297	37.36%	
No. of employes	more than 150	175	22.01%	795
	between 25 to 150	285	35.85%	
	less than 25	335	42.14%	
Average revenue	more than 10 million	75	9.43%	795
	between 5 to 10 million	280	35.22%	
	less than 5million	440	55.35%	

*Sources: authors’ accumulation (2021).*

### Estimation Strategy for Hypothesis Testing

Upon completion of data collection, descriptive statistics will be used as data analysis techniques, and the research yields quantitative data from organized and structured items. Coding will be accomplished for the structured items. After the completed questionnaire, data will be analyzed using Microsoft Excel (Microsoft for Windows, United States), SPSS (IBM, United Kingdom), and PLS-SEM-Smart PLS 3.2.7 (SmartPLS GmbH, www.smartpls.com). Among the essential viewpoints that must be view as while figuring or assessing a particular instrument are a validity and reliability. The study initiated instrumental validation for confirming the internal consistency of the considered latent constructs for measuring the variables. The reliability of the research sample has been assessed by implementing the coefficient of average variance explained (AVE) and consistency ratio (CR). The study implemented Fornell and Larcker (Fornell and Larcker, 1981) and Hetreotrait-Monotrait (Henseler et al., 2015) tests for evaluating construct discriminate validity. The efficiency of measurement models has been assessed by performing the confirmatory factor analysis with several goodness fit indexes. The prime target of hypothesis testing with both direct and indirect effects is by implementing structural equation modeling. Furthermore, the study has implemented the Hierarchical regression analysis in gauging the target variables’ effects with the incorporation of control variables in the model.

## Model Estimation and Interpretation

### Instrument Validation

Before implementing the target causal model, the stuns initiated latent construct validation by performing explanatory factor analysis to detect the possible outlier and multicollinearity. The results of instrument validation reports are in Table 4. According to standard guidelines, the factor loading coefficient in any instrument should be greater than 0.7 to be treated as suitable for further causal effects assessment (Pu et al., 2021). Furthermore, regarding the multicollinearity issue, the coefficient of variance inflation factors should be less than 3. In any circumstance, the value of VIF greater than 3 should pay more attention to the final model assessment because multicollinearity in construct can produce spurious estimation. Refers to the factor loading coefficients for the variables latent construct, it is revealed that all the factor loading coefficients are more significant than the standard thresholds, suggesting internal consistency and construction validation. Moreover, the multicollinearity test established that latent constructs are free from that issue. Considering the instruments’ validation coefficients, it is now revealed that instruments can address and detect the causal effects.

**TABLE 4:** Outer loading and multicollinearity.

	WoE	WeD	AtF	TkH	FiL	VIF
*WoE1:* access to and control over the key economic and financial assets	0.835					1.5196
*WoE2:* access to decent work and control over the work related decision	0.853					1.3654
*WoE3:* capabilities, household relations, gender unbiased business environment	0.894					2.4657
*WoE4:* Individual capabilities in relation to access and control over assets and jobs	0.917					1.1853
*WoE5:* Legal protection and reform of discriminatory laws and regulations	0.882					2.8033
*WoE6:* Policies to promote workplace equality (e.g., including equality in work hours, conditions, and wages)	0.859					1.2065
*WeD1:* Interested in experiencing new activities		0.884				1.5294
*WeD2:*Utilizing the opportunities		0.919				2.71
*WeD1:*Helping to improve the society		0.869				2.7213
*WeD3:*Improving the social status		0.95				1.4729
*WeD4:*Providing innovations		0.846				1.2612
*AtF1*:Lack of ability to draw a business plan			0.908			2.7544
*AtF2:* Higher interest rate			0.886			2.7006
*AtF3:* Lack of collateral of assets			0.989			2.7763
*AtF4:* Short duration for repayment of loan			0.929			1.5659
*AtF1:* Lengthy banking process			0.886			1.6421
*TkH1:* Proud to support sustainable technology implementation				0.922		1.7591
*TkH2:* positive toward sustainable technology adoption				0.876		1.833
*TkH3:* sustainable technology can help improve the environment				0.866		2.9047
*TkH4:* sustainable technology can contribute to the SMEs development				0.904		2.5876
*TkH5:* agree with the idea of sustainable technology				0.958		1.1206
*FiLt1*: Use authorized, arranged overdraft or line of credit					0.972	2.2256
*FiLt2:*Take out a personal loan from a financial service provider					0.893	1.446
*FiLt3:*Before buying something, I ask myself if I have paid my necessary expenses					0.865	1.4727
*FiLt4:*Before signing a financial contract, I carefully read its contents					0.928	1.147

*WoE, women empowerment; WeD, women entrepreneur development; AtF, access to finance; TkH, technical know-how; FiL, financial literacy.*

Table 5 shows the descriptive assessment of latent constructs of the causal model. It reveals that the mean and *SD* of access to finance is 3.3422 (*SD* = 1.1090), mean and *SD* of financial literacy is 3.3424 (*SD* = 1.26901). Technological know-how’s mean and *SD* is 3.3219 (*SD* = 1.20124), while the mean and *SD* of women entrepreneurs in SMEs is 3.2119 (*SD* = 1.12196). The mean and *SD* of empowerment of women is 3.2917 (*SD* = 1.21075).

**TABLE 5:** Descriptive statistics of constructs.

	Min		Max		Mean	Std. Dev
**Panel A: descriptive statistics**						
Access to Finance	1.50		5.00		3.3422	1.10900
Financial Literacy	1.00		5.00		3.3424	1.26901
Technological Know-how	1.60		5.00		3.3219	1.20124
Women Entrepreneurs Development	1.00		5.00		3.2119	1.12176
Empowerment of women	1.50		5.00		3.2917	1.21075
**Panel B: Pair-Wise Correlation**						

**Variables**	**AtF**	**FSS**		**TI**	**WeD**	**WoE**

Access to Finance	1					
Financial Literacy	0.711**	0.631**				
Technological Know-how	0.762**	0.638**		1		
Women Entrepreneurs Development	0.666**	0.760**		0.621**	1	
Empowerment of women	0.661**	0.672**		0.660**	0.663**	1

**significant level at a 5%.

Next, the results of latent construct reliability and convergence validity are displayed in Table 6. According to the standard threshold for instruments reliability, it should be greater than 0.7 for Cronbach’s Alpha, rho_A, and CR, and the coefficient of AVE should be greater than 0.5 (Sarstedt et al., 2019). Study findings divulged that all the coefficients in assessing the reliability of the constructs had been exposed with a higher degree of precision; that is, the estimated coefficients are greater than the standard thresholds.

**TABLE 6:** Reliability of constructs.

	Cronbach’s Alpha	rho_A	CR	AVE
Access to Finance	0.944	0.922	0.931	0.844
Financial Literacy	0.904	0.871	0.874	0.818
Technological Know-how	0.895	0.866	0.892	0.83
Women Entrepreneurs Development	0.892	0.876	0.897	0.816
Empowerment of women	0.933	0.845	0.898	0.841

The study implemented Fornell and Larcker (Fornell and Larcker, 1981) and Hetreotrait-Monotrait (Henseler et al., 2015) tests to evaluate the construct discriminate validity. The results of discriminant validity reports are shown in Table 7. According to Fornell and Larcker test, it is revealed that the diagonal coefficients are higher than the value of internal correlation coefficients. Furthermore, the test statistics of HTMT are less than the standard threshold that is <0.85. Study findings established the absence of discriminant validity in the data set.

**TABLE 7:** Discriminant validity.

Constructs	Fornell and Larcker					Hetreotrait-Monotrait				
	WoE	WeD	AtF	TkH	FiL	WoE	WeD	AtF	TkH	FiL
WoE	0.9186					1				
WeD	0.713	0.9044				0.663	1			
AtF	0.61	0.671	0.9110			0.799	0.673	1		
TkH	0.746	0.678	0.74	0.9033		0.632	0.797	0.82	1	
FiL	0.635	0.684	0.691	0.628	0.9170	0.817	0.82	0.698	0.82	1

*WoE, women empowerment; WeD, women entrepreneur development; AtF, access to finance; TkH, technical know-how; FiL, financial literacy.*

### Measurement and Structural Model Estimation

The result of the measurement model displays in Table 8. Refers to measurement model goodness of fit test, it is observed that test statistics of normed fit index (NFI) (0.996), relative fit index (RFI) (0.915), goodness of fit index (GFI) (0.919), incremental fit index (IFI) (0.942), comparative fit index (CFI) (0.982), and non-normed fit index (NNFI, also known as TLI) (0.992), which is greater than the standard thresholds that are 0.9. These findings suggest that the latent construct for measurement variables is internally consistent and can produce an efficient estimator. For parsimonious assessment, it is found that test statistic is less than 3. According to Arbuckle (2011), the threshold ratio has to be between 1 and 3. Since it is apparent that most goodness fit indexes are higher than the standard value for model acceptability, one can conclude the well-fitted model construction.

**TABLE 8:** Results of measurement model estimation.

	Υ	R^2^	CR	AVE	The goodness of fit index	
WoE1	0.954	0.910	0.952	0.769	X^2^ = 25.6142 (*p* = 0.00)	
WoE2	0.929	0.863			NFI=	0.996
WoE3	0.86	0.740			RFI=	0.915
WoE4	0.927	0.859			GFI=	0.919
WoE5	0.854	0.729			IFI=	0.942
WoE6	0.902	0.814				
WeD1	0.938	0.880	0.914	0.816	CFI=	0.982
WeD2	0.956	0.914			TLI=	0.992
WeD3	0.922	0.850			RMSEA=	0.01
WeD4	0.856	0.733				
WeD5	0.841	0.707				
AtF1	0.923	0.852	0.916	0.823		
AtF2	0.901	0.812				
AtF3	0.872	0.760				
AtF4	0.903	0.815				
AtF5	0.938	0.880				
TkH1	0.889	0.790	0.822	0.805		
TkH2	0.922	0.850				
TkH3	0.881	0.776				
TkH4	0.898	0.806				

*WoE, women empowerment; WeD, women entrepreneur development; AtF, access to finance; TkH, technical know-how; FiL, financial literacy.*

Next, the study performed structural equation modeling for evaluating the mediation effects of access to finance, technological know-how, and financial literacy through women entrepreneur development. The structural model results displayed in Table 9 consist of two data representation panels: panel A for direct effect and Panel B for indirect effects.

**TABLE 9:** Hypothesis testing with structural model.

Relationships	Path	Std. Dev.	*t*-value	*p*-value	Goodness of fit-index
***Panel-A: Direct effects***					
AtF → WeD	0.248***	0.055	4.509091	0	(*p* = 0.00)
AF → WoE	0.166***	0.067	2.477612	0	RFI = 0.895
TkH WeD	0.184***	0.068	2.705882	0.005	GFI = 0.983
TkH → WoE	0.138***	0.067	2.059701	0.023	IFI = 0.938
FiL → WeD	0.159***	0.066	2.409091	0.003	CFI = 0.938
FiL → WoE	0.227***	0.057	3.982456	0	TLI = 0.922
WoD → WoE	0.215***	0.065	3.307692	0	RMSEA = 0.03
***Panel-B: Indirect effects***					AIC = 312.01
AtF → WoD WoE	0.157***	0.074	2.121622	0.003	PNFI = 0.723
TkH - > WoD - > WoE	0.08***	0.055	1.454545	0.036	PCFI = 0.745
FiL - > WoD - > WoE	0.113***	0.061	1.852459	0.001	$X_{62}^{2}=228.01$

*AtF, access to finance; FSS, family and social support; EI, educational issues; ATM, access to market network; TI, technological issues; SI, social issues; WESMEs, women entrepreneurs in SMEs; EW, empowerment of women.*

The superscripts *** denotes the level of significance at a 1% level of significant.

*Source: Smart PLS Output.*

Refers to direct effects from access to finance, technological know-how, and financial literacy on women entrepreneur development (women empowerment). The study documented a positive association between access to finance and women entrepreneur development (women empowerment), a coefficient of 0.248, *P* < 0.01 (0.166, *P* < 0.01), which is supported by existing literature such as Manwari et al. (2017); Arora (2021). The study findings suggest that the external credit facility has played a catalyst role in augmenting the state of women empowerment and entrepreneur development, implying that venture capital sources motivate women to enter the process of entrepreneur development, especially those who are scared of inadequate capital support. In a study of Ghosh and Neogi (2017), they advocated that financial services accessibility, especially women in the economy by offering micro or specialized credit, open an avenue for entrepreneurship development. It is because of limited economic resources and financial constraints that dwindle the speed of entrepreneur development activities.

The role of technological understanding has established a positive linkage with women entrepreneur development (women empowerment) with a coefficient of 0.184, *P* < 0.01 (0.138, *P* < 0.01), which align in the existing literature such as Prljić et al. (2015). Study findings suggest that technical expertise assists in growing entrepreneurship development among women and eventually being women’s economic and financial capacity, commonly known as empowerment.

Refers to financial literacy effects on women’s entrepreneurial development and empowerment, the study revealed a positive statistically significant association with women entrepreneur development (women empowerment), a coefficient of 0.159; *P* < 0.01 (0.227; *P* < 0.01). Study findings postulated that financial literacy increase women entrepreneurial development in women and support sustainability in their operation, which is aligned with the existing literature such as Sucuahi (2013); Wanambisi and Bwisa (2013), Abubakar (2015); Atandi et al. (2017), Kuruvilla and Harikumar (2018). Financial literacy is the capacity to properly manage financial resources throughout the business life cycle, interact with financial goods and services, and recognize and use financial management effectively.

It presents the results of three mediation models that have been extracted. Access to finance has illustrated from the table that both direct and indirect relationship is significant for AtF⇢FL⇢WoE mediation relation. However, the indirect to total effects ratio is below 0.50, indicating full mediation (Hair et al., 2016; Ali et al., 2020; Tabassum and Amin, 2021). The findings unfold that Women Entrepreneurs in SMEs did not completely suppress the Women Entrepreneurs in SMEs on the Empowerment of women and the magnitude of direct and indirect effects are close to each other. It indicates the importance of Women Entrepreneurs in SMEs ‘ usefulness in influencing the trusting behavior of entrepreneurs. These findings are by McKnight et al. (2002), who have found a significant impact of Access to Finance and Women Entrepreneurs in SMEs on women’s empowerment. Women Entrepreneurs in SMEs mediate between Access to Finance and Empowerment, and women have an effect size above 0.5. However, the indirect effect is below 4, and it has partial mediation between them. Also, it reveals that both direct and indirect effects are significant for FSS⇢TI⇢WoD mediation model. However, the ratio of indirect to total effect is above 0.5, indicating full mediation.

### Multivariate Analysis

The multivariate hierarchical regression analysis results are displayed in Table 10 with the dependent variable of women empowerment, table with women entrepreneur development as a dependent variable, and table with women empowerment through women entrepreneur development.

**TABLE 10:** Hierarchical regression analysis.

	Model (1)	Model (2)	Model (3)
**Panel –A: women entrepreneurship sustainability**			
AtF	0.1273*** (0.0321) [3.9657]	0.2407*** (0.0234) [10.2863]	0.163*** (0.0299) [5.4515]
TkH	0.3086*** (0.0153) [20.1699]	0.1497*** (0.0329) [4.5501]	0.1527*** (0.0338) [4.5177]
FiL	0.1338*** (0.0144) [9.2916]	0.2266*** (0.0205) [11.0536]	0.2926*** (0.0147) [19.9047]
GS		0.1401*** (0.0308) [4.5487]	0.2533*** (0.0248) [10.2137]
SME training		0.3008*** (0.0303) [9.9273]	0.2166*** (0.0208) [10.4134]
SS		0.1527*** (0.0136) [11.2279]	0.3129*** (0.0259) [12.081]
FS		0.234*** (0.0182) [12.8571]	0.3186*** (0.0322) [9.8944]
Constant	0.2361*** (0.0261) [9.0459]	0.1752*** (0.0153) [11.4509]	0.1401*** (0.0135) [10.3777]
R2	0.4911	0.5847	0.7287
Adj.R	0.6032	0.8054	0.8893
Change	0.1121	0.2207	0.1606
D-W stat	2.538	2.446	2.386
**Panel –B: women entrepreneurship Empowerment**			
AtF	0.1206*** (0.0275) [4.3854]	0.2446*** (0.0158) [15.481]	0.221*** (0.0317) [6.9716]
TkH	0.1893*** (0.017) [11.1352]	0.1401*** (0.0185) [7.5729]	0.3038*** (0.0262) [11.5954]
FiL	0.1628*** (0.0263) [6.1901]	0.3246*** (0.0148) [21.9324]	0.1411*** (0.0289) [4.8823]
GS		0.1998*** (0.0333) [6.001]	0.1462*** (0.0267) [5.4756]
SME training		0.1223*** (0.032) [3.8218]	0.1877*** (0.0126) [14.8968]
SS			0.1515*** (0.0219) [6.9178]
FS			0.3202*** (0.0285) [11.235]
Constant	0.3186*** (0.0334) [9.5389]	0.1314*** (0.0179) [7.3407]	0.2077*** (0.0299) [6.9464]
**R2**	0.5148	0.6586	0.760
**Adj.R**	0.6265	0.819	0.8845
**Change**	−0.1117	0.1604	0.1236
D-W stat	2.32	2.483	2.512
**Panel –C: women entrepreneurship Empowerment through entrepreneurship sustainability**			
AtF	0.2539*** (0.0255) [9.9568]	0.3314*** (0.0224) [14.7946]	0.3248*** (0.0151) [21.5099]
TkH	0.1382*** (0.0243) [5.6872]	0.3242*** (0.0248) [13.0725]	0.2098*** (0.0219) [9.5799]
FiL	0.3325*** (0.0244) [13.627]	0.3248*** (0.0206) [15.7669]	0.2013*** (0.0185) [10.881]
GS		0.1564*** (0.0219) [7.1415]	0.2541*** (0.0152) [16.7171]
SME training		0.324*** (0.0228) [14.2105]	0.2167*** (0.0328) [6.6067]
SS			0.2533*** (0.0267) [9.4868]
FS			0.2907*** (0.0151) [19.2516]
Constant	0.1461*** (0.0201) [7.2686]	0.2549*** (0.0254) [10.0354]	0.2829*** (0.033) [8.5727]
**R2**	0.4984	0.6278	0.694
**Adj.R**	0.5666	0.7763	0.8882
**Change**	−0.0682	0.1485	0.1942
D-W stat	2.396	2.479	2.407

*AtF, access to finance; FS, family support; SS, social security; GS, government support: FiL, Financial literacy; TkH, technical know-how; WeD, women entrepreneurs development; WoE, women empowerments.*

The superscripts *** denotes the level of significance at a 1% level of significant.

*Note that the values in () represents a standard error, and the value in [] represents the standard error of the coefficients.*

Considering the model output with entrepreneurship sustainability see panel-A, it is apparent that access to finance exposed a positive statistically significant association with entrepreneurship development in all three model estimations. More specifically, a 10% growth in external credit facilities for women entrepreneurs increases sustainable development prospects by 1.27 to 2.403%. Study findings suggest that business expansion and development can be accelerated by supporting the scope of credit accessibility. The effects of technological know-how on WeD were documented positive and statistically significant at a 1% level. Studies suggest that operational efficiency with advanced technology integration and application assists in establishing sustainability in entrepreneurial development. In particular, a 10% improvement in technological knowledge in women entrepreneurs can increase the women entrepreneur sustain development by 1.497 to 3.086%. In addition, the positive effects of financial literacy on WeD were revealed, implying that financial knowledge enables women to select the appropriate means of fund management and optimal economic resources allocation. This results in sustainable development with the optimal application of sources of financing and the best use of available financial resources. Considering the list of control variables: government support, SMEs training, social security, and family support has revealed a positive statistically significant role in women entrepreneurial sustainable development.

Next, model estimation with women empowerment as a dependent variable in the equation and estimated coefficients are displayed in panel-B. study findings revealed that access to financial benefits in the financial sector positively accelerates women’s empowerment. More precisely, a 10% improvement in women’s accessibility to the formal financial offering can augment women empowerment by 1.206 to 2.446%. Findings suggest that available financing avenues enable women to expand their earning capacity, which eventually supports them in establishing economic and financial empowerment. Furthermore, the state of women empowerment in Bangladesh has also accelerated with improved technological knowledge and financial literacy among women, especially women entrepreneurs. In particular, a 10% development in technical knowledge (financial literacy) results in developing the present state of women empowerment by 1.42 to 3.048% (1.412 to 3.148%). The study established that women habituated with IT application and integration enable them to reach operational efficiency, whereas financial literacy opened their minds in selecting the appropriate means of financial offerings as well as resources optimization.

## Discussion

The study has gauged the role of access to finance, technological know-how, and financial literacy on women’s empowerment through women’s entrepreneurial development in Bangladesh. Study findings assess the causal effects in three dimensions: the role of an independent variable on women entrepreneurial progress, second the target variables’ effects on women empowerment, and third the effects on women empowerment through women entrepreneur sustainability that is the mediation effects.

First, study findings divulged positive statistically significant linkage between target variables, i.e., access to finance, technical know-how, financial literacy, and women entrepreneur sustainability. Study findings suggest that to bring prosperity to women’s entrepreneurial development, it is essential to ensure easy access to financial services and credit extension programs, opportunities for enhancing technical expertise, and financial education. In a study, Mungai (2021) postulated that women entrepreneurs are passing with several building blocks in entrepreneurial capacity development among factors the access to credit placed in the apex spot. Accessibility to financial products and services inject required forces in entrepreneur development by subsiding the progress of economic and resource constraints. Access to finance has opened up an economic avenue for women’s entrepreneurial development (Manwari et al., 2017). A study was conducted by Khaleque (2018) investigating the role of credit expansion facilities on the performance of women entrepreneurs. The study documented that access to credit facilities matters for women entrepreneurial development that increases revenue and profit sustainability has achieved.

Lack of Access to financing continues to be a barrier to entrepreneurship for men and women alike, although research indicates that women face higher obstacles. A significant barrier cited by women entrepreneurs is a lack of institutional financing for start-ups and firms’ growth. Financial institutions may tap into a rising market of women customers by offering well-designed microfinance, SME, and leasing products that suit their requirements. Similarly, increasing women’s demand for money requires a combination of financial goods and well-thought-out regulations. This must involve increasing women’s ownership and control of their land and assets (i.e., property rights) to assist them in meeting collateral requirements and increasing women’s financial knowledge to utilize current financing programs. Additionally, policymakers should support company development services that assist women in gaining access to bank financing.

Second, the technological know-how effects on women’s entrepreneurial development and empowerment and study revealed a positive association. Study findings suggest that entrepreneurial growth in the dynamic environment is essential to have technical expertise for exploiting the advancement in operational efficiency through technological integration. The potential for women entrepreneurship to grow due to IT integration to enhance company operations and expand market share. IT integration enables businesses to remain accessible 24 h a day to customers worldwide and customize services. This helps create new entrepreneurial possibilities, particularly for women-led businesses. According to Costanza et al. (2003), women’s entrepreneurship contributes to economic development and provides job possibilities. Numerous research has indicated a link between women entrepreneurship and women empowerment. Empowerment is the process by which an individual move from a position of powerlessness to one of relative control over his or her life, destiny, and surroundings. This shift may be seen in both an increase in perceived capacity to control and an increase in actual ability to control.

Third, the role of financial literacy on women entrepreneurial development and women empowerment and in this study established positive statistically significant linkage between them. It suggests from study findings that financial understanding allows appropriate selection of credit extension model and substantially reduces the risk of a future failure. According to Engström and McKelvie (2017), more financial knowledge improves financial decision-making and an increased appreciation for financial problems faced in the present or future. Additionally, Lusardi and Tufano (2015) discovered a stronger correlation between financial knowledge and financial literacy. This demonstrates the critical importance of understanding financial terminology and ideas, the foundation for good financial decision-making. As a result, the more one’s financial literacy, the greater one’s literacy level.

Additionally, Natoli (2018) found a significant correlation between financial literacy and an individual’s financial understanding and attitude. Egbo et al. (2020) investigate the association between financial literacy and access to credit by women entrepreneurs in Nigeria. The study documented that financial knowledge was a key element in expanding women-owned businesses, particularly during start-up. Additionally, the study established that financial expertise is essential to the development and success of women-owned businesses. Financial literacy and ability are necessary for women to build an entrepreneurial spirit and grow their entrepreneurial activities effectively, manage their personal and family money, and increase their success in their entrepreneurial areas of interest. It is critical to equip women entrepreneurs with the knowledge and skills necessary to make smart and good financial decisions (Lafortune and Tessada, 2015). Further, financial expertise is essential to developing and succeeding women-owned businesses, fundamental accounting, and bookkeeping skills, budgeting, financial management, money raising and allocation, loan management, investment, and people management. A lack of financial skills makes acquiring and managing capital/resources difficult for women company owners.

## Conclusion and Policy Suggestions

Women entrepreneurship, according to Nimalathasan (2010), is a byproduct of women’s empowerment. Entrepreneurship has the potential to empower women personally and economically. According to Islam et al. (2018), women’s entrepreneurship development and empowerment are mutually reinforcing. Women entrepreneurs are more empowered in social, economic, and cultural domains. Women’s participation in business endeavors has boosted their influence and exposure to decision-making inside and beyond the family.

The study demonstrated that accomplishing women empowerment through Women entrepreneurship development is one alternative but has to put considerable interest in offering efficient management of key determents for WeD such as access to external financing scope, advanced technological integration, and financial understanding. Moreover, starting and looking after self-business appears to be trying for women as it builds the burden of obligations regarding women in both home and working environments. Furthermore, while turning into influential entrepreneurs, women face different social difficulties from their relatives and society. Every business needs finance to start a business. Women have issues accessing advances from banks, money related organizations. They have too restricted in various SME divisions other than traditional organizations. Besides, because of the absence of technological skills, they cannot deal with their records appropriately. Furthermore, women entrepreneurs in Bangladesh face various social, political, and marketing challenges in starting and keeping up their organizations. Thus, as a rule, they stay stuck in the beginning period of business development.

Thus, appropriate measures should be taken to beat the difficulties SME women entrepreneurs face, and the support of institutions and government is significant. These may incorporate coordinated money-related strategy, appropriate business education, technical help and training, improved laws circumstance in Islamic perspective, logistics help, and local items’ progress – employment increase economic growth in Bangladesh. Moreover, policies and procedures should assure the equivalent investment of men and women in life for progression. The administration, NGOs, and different business associations can sort out a few critical projects to help women entrepreneurs in SMEs.

We come up with the following policies suggestion for ensuring entrepreneurship sustainable development and women empowerment in the economy:

(a) Women consumers are usually self-employed and responsible for home costs, food, and child care, particularly susceptible to taking on loans with strict payback plans. Institutions seeking to expand their women client base should develop novel loan products (particularly those that address lifecycle requirements) and marketing campaigns (for example, through sponsorship of non-financial services and rewarding referral systems). Training bank employes about the unique requirements of prospective (and current) women clients may be a critical first step toward expanding the women’s. Furthermore, financial institutions may seek better processes and more flexible payback plans to meet customer needs better. As well as promoting women’s savings in various nations, financial institutions have also encouraged lending to women via group lending and guarantee programs.
(b) Compared to enterprises run by males, women entrepreneurs cannot deposit collateral to get loans from formal institutions. Thus leasing is a suitable financing option for them. The importance of leasing in Bangladesh is that it is asset-based finance that is the equipment itself is a security, the institution maintains control of the asset. At the same time, the leasing company has an agreement to use it as long as needed. Women small- and medium-sized enterprise (SME) owners often have trouble getting funding from traditional financial institutions due to a lack of adequate operating cash, credit history, and collateral resources. Women-owned microenterprises may particularly benefit from leasing. Small- and medium-sized enterprises (SMEs) cannot get loans for equipment financing because they do not have the collateral required by banks.

(c) Women-owned companies may benefit from the financial performance of services supplied by the government, NGOs, and other interest groups. It has enabled many of its nine hundred members to get financing by allowing them to submit loan applications and business registrations, tax return forms, and audited financial statements.
(d) Women entrepreneurs could improve their financial literacy skills on their own by attending seminars and courses on financial literacy and reading about money. Their lifestyles would be enriched and improved as a result of increased financial knowledge. Simultaneously, financial literacy teaches students how to manage their financial situations and meet their financial responsibilities on time and at the lowest possible cost. Financial literacy will increase their chances of company success and their capacity to manage it efficiently and enhance it. Additionally, students will learn more about saving and investing to diversify their sources of income and adapt to the changing financial environment. Finally, financial possibilities offered by this new financial age should be identified, and micro-businesses should pay them greater attention.

This study’s real constraint is that it only focuses on women entrepreneurs’ difficulties in Dhaka, which shows small-scale research. Future research can be in other cities by covering a large portion of the respondents of this study was involved with comparative sort of organizations. In this manner, diverse encounters from various organizations are missing here to comprehend the issues women entrepreneurs in Bangladesh face. Future research can be conducted in other specific areas of business.

## Data Availability Statement

The raw data supporting the conclusions of this article will be made available by the authors, without undue reservation.

## Author Contributions

AA contributed to introduction, methodology, and first draft preparation. MQ contributed to introduction, methodology, empirical model estimation, and final preparation. Both authors contributed to the article and approved the submitted version.

## Conflict of Interest

The authors declare that the research was conducted in the absence of any commercial or financial relationships that could be construed as a potential conflict of interest.

## Publisher’s Note

All claims expressed in this article are solely those of the authors and do not necessarily represent those of their affiliated organizations, or those of the publisher, the editors and the reviewers. Any product that may be evaluated in this article, or claim that may be made by its manufacturer, is not guaranteed or endorsed by the publisher.
